# Ubiquitous Transgene Expression of the Glucosylceramide-Synthesizing Enzyme Accelerates Glucosylceramide Accumulation and Storage Cells in a Gaucher Disease Mouse Model

**DOI:** 10.1371/journal.pone.0116023

**Published:** 2014-12-31

**Authors:** Sonya Barnes, You-Hai Xu, Wujuan Zhang, Benjamin Liou, Kenneth D. R. Setchell, Liming Bao, Gregory A. Grabowski, Ying Sun

**Affiliations:** 1 The Division of Human Genetics, Cincinnati Children's Hospital Medical Center, Cincinnati, Ohio, United States of America; 2 The Division of Pathology and Laboratory Medicine, Cincinnati Children's Hospital Medical Center, Cincinnati, Ohio, United States of America; 3 The Department of Pediatrics, University of Cincinnati College of Medicine, Cincinnati, Ohio, United States of America; 4 Dartmouth-Hitchcock Medical Center, Geisel School of Medicine, Dartmouth College, Lebanon, New Hampshire, United States of America; 5 Synageva BioPharma Corp., Lexington, Massachusetts, United States of America; University Hospital S. Maria della Misericordia, Udine, Italy

## Abstract

Gaucher disease is a lysosomal storage disease caused by defective activity of acid β-glucosidase (GCase), which leads to the accumulation of its major substrates, glucosylceramide (GlcCer) and glucosylsphingosine (GlcSph) in many cells. To modulate cellular substrate concentration in viable mouse models of Gaucher disease (*Gba1* mutants), a novel mouse model was created with enhanced glycosphingolipid biosynthesis. This was accomplished by cross-breeding *Gba1* mutant mice with mice expressing a transgene (GCStg) containing the mouse glucosylceramide synthase (GCS, *Ugcg*) cDNA driven by the ROSA promoter, yielding GCStg/*Gba1* mice. The GCStg rescued *Ugcg* null mice from embryonic lethality. GCStg/*Gba1* mice showed 2–3 fold increases in tissue GCS activity as well as accelerated GlcCer accumulation and the appearance of lipid-laden CD68 positive macrophages in visceral organs. Although GlcCer/GlcSph concentrations were elevated in the brain, there was no neurodegenerative phenotype up to 1 yr of age conceivably due to the greater residual GCase hydrolytic activity in the brains than in the visceral tissues of 9V/null mice. These studies provide ‘proof of principle’ for threshold substrate flux that modifies phenotypic development in Gaucher disease and other lysosomal storage diseases.

## Introduction

Gaucher disease is an autosomal recessive disorder resulting from defective catalytic activity of the lysosomal enzyme, acid β-glucosidase [GCase. glucocerebrosidase, E.C.3.2.1.45]. More than 300 mutations have been characterized in the *GBA1* locus as causal to GCase defects [1]. Insufficient GCase activity leads to progressive accumulation of its uncleaved substrates, mainly glucosylceramide (GlcCer) and glucosylsphingosine (GlcSph), and a continuum of clinical phenotypes. The presence of lipid-laden macrophages or ‘Gaucher cells’ in visceral organs is the pathologic hallmark of this disease [1]. Gaucher disease is classified into three types. The most common form in the Western world, Type 1, has visceral involvement; types 2 and 3 have central nervous system (CNS) involvement and are more common outside of the Western world [1]–[9]. The CNS pathogenesis is non-phagocytic and results in neuronal death/drop-out that is thought to be propagated by inflammation and accumulation of the toxic substrates, GlcSph and/or GlcCer [4], [10].

Mouse models of lysosomal storage disease have been indispensable in elucidating the relationship between gene defects and disease manifestation. There are no spontaneously occurring *Gba1* mutant mouse models. The *Gba1* null mouse dies within 24 hrs after birth due to defects in skin permeability [11]. Severe neuronopathic Gaucher disease mice were generated and showed rapid development of gross phenotypes and died by day 14 [12], [13]. The short lifespan of this neuronopathic model (similar to type 2 GD in human) limits their utility for elucidating the pathophysiologic mechanisms of chronic visceral Gaucher disease. Viable Gaucher disease mouse models have been created by ‘knocking in’ various point mutations flanked with *loxP* sequences and inserted in the mouse *Gba1* locus encoding GCase including V394L, D409H, D409V, or D409V/null [14]. Viable L444P homozygous mice are generated by interbreeding the double heterozygous mice (*Gba*+/L444P; *Ugcg*+/KO) for *Gba1* L444P/L444P; *Ugcg*+/+ pups [15] or by point mutation insertion flanked with *loxP* sequences [16]. GCase *in vitro* activities in these mice are about 2.5–10% of wild type (WT) level in viscera (liver, lung, and spleen) and about 21–28% of WT level in the CNS [14], [15]. Homozygotes for these point mutations, except for N370S, have normal life spans (1∼2 years) and reproduction. [14]–[16]. However, an abnormal neurological phenotype has been determined in the mouse model associated with D409V mutation that presents α-synuclein pathology concomitant with memory deficit at one year of age [17]. Compared to the human phenotypes, attenuated diseases are in the mouse models that were seen in other lysosomal storage disorders e.g., α-galactosidase A and β-hexosaminidase A-deficient mouse models for Fabry and Tay-Sachs disease, respectively [18]. In the Tay-Sachs disease model, this was explained by species specificities of metabolic pathways [18], [19]. For Fabry and Gaucher disease mouse models, the molecular basis for the unexpectedly mild mouse phenotypes is unknown. Developing a rapidly progressive mouse models with continuously increasing GlcCer in tissues to accelerate the gross phenotypes could expedite pathogenesis studies.

GlcCer is synthesized via UDP-glucose:ceramide glucosyltransferase or glucosylceramide synthase (GCS) [20], [21] and is encoded by *Ugcg* [22], [23]. GCS has a central role in organ development since GlcCer is the precursor for over 300 different glycosphingolipids that are needed for mouse embryonic development beyond the preimplantation eight-cell stage (E2.5–E3.0) [24]. *Ugcg* knockout mice exhibit embryonic lethality with development proceeding to E6.5 and then massive apoptosis leading to complete resorption by E8.5 [24]. Neural cell depletion of *Ugcg* leads to glycosphingolipids deficiency followed by down-regulation of genes involved in brain development and homeostasis [25]. The neuronal expression of GCS has implications on autophagy flux [26], and affect obesity and energy homeostasis [27]. GCS is an integral membrane protein of the cis/medial Golgi that catalyzes the transfer of a β-glucose to ceramide forming GlcCer [28]–[30]. The catalytic domain of GCS is located on the cytosolic side of the Golgi requiring GlcCer to be flipped to the luminal side in order to undergo subsequent modifications to make high order glycosphingolipids by other glycosyltransferases [31]–[35].

Controlling *in vivo* GlcCer synthesis can modulate substrate level in glycosphingolipids storage disease mice and change the disease outcome, e.g., pharmacological inhibition of GCS as in substrate reduction therapy (SRT) in the mouse models of Gaucher disease [36], Sandhoff disease [37], Fabry disease [38] and GM1 gangliosidosis [39]. Alternatively, an integrated and highly expressed GCS from the transgene might be able to promote GlcCer synthesis to the level above the threshold that would lead to accelerated phenotypic development. This has been demonstrated by enhanced synthesis of sulfatide in the arylsulfatase (ASA)-deficient mouse model that transformed a mild phenotype into mimic of the more aggressive human disease [40]. Overexpression of the mouse *Gal3st1* cDNA (galactosylceramide sulfotransferase) in oligodendrocytes and Schwann cells of arylsulfatase A (ASA) deficient mice increased sulfatide storage in the nervous system and development of a myelin pathology in the CNS, particularly in the peripheral nervous system (PNS) similar to metachromatic leukodystrophy patients [41]. From these studies, we hypothesized that increases of GlcCer synthesis would accelerate phenotype progression in Gaucher disease mutant mice. With deficient GCase hydrolytic activity for GlcCer degradation, increased GlcCer synthesis could be an approach to ‘tip the scale’ to rapidly reach substrate threshold concentrations that speed up manifestations of visceral and/or neurological lesions in Gaucher disease. GlcSph is quantitatively a minor accumulated substrate in Gaucher disease [4], [10]. The source of GlcSph has been suggested to be from GlcCer after deacylation by acid ceramidase [42], therefore GlcSph concentrations would directly correlate with GlcCer production and simultaneously increase in Gaucher disease. Thus, high expression of GCS transgene would lead to increases of both substrates, GlcCer and GlcSph.

Here, a GCS cDNA transgene (GCStg) driven by the ubiquitous ROSA promoter was used to increase the substrate threshold concentration in our 9V/9V and 9V/null *Gba1* mutant mice. The tissue lipid concentrations and histology were analyzed at various ages to evaluate the effect of GCStg in accelerating substrate concentration and Gaucher disease manifestations. The results from this study will be useful as a ‘proof of principle’ to determine the threshold level of substrate and/or flux that influences phenotypic development in Gaucher and other lysosomal storage diseases.

## Materials and Methods

### Materials

The following were from commercial sources: Trizol™ reagent, Superscript™ First-Strand Synthesis System for RT-PCR Kit, NuPAGE 4–12% Bis-Tris gel, and NuPAGE MES SDS running buffer, N-6-[(7-nitrobenzo-2-oxa-1, 3-diazol-4-yl) amino] hexanoyl-4-d-erythro-sphingosine (C6-NBD-ceramide), Alexa-Fluor555 (Invitrogen, Carlsbad, CA); anti-β-actin monoclonal antibody (Sigma, St. Louis, MO); rat anti-mouse CD68 monoclonal antibody (Serotec, Oxford, UK); M-PER Mammalian Membrane Protein Extraction Reagent and BCA Protein Assay Reagent (Pierce, Rockford, IL); [32P] dCTP (DuPont-New England Nuclear Research Products, Boston, MA); Molecular Dynamics Storm 860 scanner, Hybond™-ECL™ nitrocellulose membrane, and ECL detection reagent (Amersham Biosciences, Piscataway, NJ); antifade/4,6-diamidino-2-phenylindole (DAPI) (Vector Laboratory, Burlingame, CA); FITC-conjugated goat anti-rabbit antibody and rhodamine-conjugated goat anti-rat antibodies (ICN Biomedicals, Aurora, OH); pBROAD3-mcs vector carrying ubiquitous murine ROSA26 promoter (InvivoGen, San Diego, CA); Genomic BAC probe RP23 (BACPAC Resources Center, Oakland CA); Nick Translation Kit (Abbott Molecular, Des Plaines, IL); Hybond N+ nylon membranes (Amersham Bioscience, Freiburg, Germany).

### Generation of transgenic mice

Mouse GCS cDNA containing the entire protein coding region was amplified from mouse brain cDNA by PCR using the oligonucleotides 5′- GAGTCTAAGCTTATGGCGCTGCTGGA-3′ (forward primer) and 5′-CGTGGGTCTAGAGTCACAGAGGTCTTC-3′ (reverse primer) which contained Hind III and Xba I restriction sites (underlined), respectively. To create the GCS transgene, amplified cDNA was subcloned into Hind III and Xba I sites of the pBROAD3-mcs vector downstream of ubiquitous murine ROSA26 promoter. The GCS transgene (1.2 kb) cDNA (GCStg), including human β-globin 3-UTR and polyadenylation signal, was released by Pac I digestion followed by agarose gel purification. Pronuclear injection into fertilized eggs from FVB mice was performed at the University of Cincinnati Transgenic Core (Cincinnati, OH). Ten independent founder lines were tested for germline transmission. 6 founders were maintained on a mixed C57BL6/129/FVB background for further characterization. Following verification of ubiquitous GCStg by RT-PCR and Northern blot, two founders (GCStg3 and GCStg9) were back-crossed into the 9V/9V and 9V/null mouse lines [14]. GCStg3 and GCStg9 were back-crossed with mice heterozygous for the *Ugcg* allele null-*Ugcg* ^ΔEX7Neo/+^ mice (a gift from Dr. Richard Proia) to generate GCStg/*Ugcg* null [24]. The mice were housed in the pathogen-free barrier facility and according to Institutional Animal Care and Use Committee (IACUC) standard procedures at Cincinnati Children's Hospital Medical Center (CCHMC). The CCHMC IACUC reviewed and approved these studies under protocol 0C02011.

### PCR genotyping

Genotyping of mice was done by PCR on genomic mouse tail DNA. For GCStg cDNA, PCR was conducted using forward primer 5′-ATTATGCTGTGGCCTGGTTC and reverse primer 5′-GACCTCCCACATTCCCTTTT-3′ to amplify a 346-bp fragment in 25 µL of PCR buffer (Invitrogen) with 3 mM MgCl_2_. Reactions were cycled 35 times as follows: 94°C for 3′, 94°C for 1 min, 56°C for 1 min and 72°C for 2 min. For GCase point mutants, the *loxP* site was used as a marker, which generated a 391 bp fragment from WT and a 485 bp fragment (391 bp+94 bp *lox-P* junction sequence in intron 8) for the mutant alleles [14]. For endogenous *Ugcg*, PCR genotyping was conducted as described [24].

### Semi-quantitative RT-PCR

Total RNA was extracted from mouse tissues using Trizol Reagent. Reverse transcription of total RNA (1 µg) was carried out using Gibco BRL Superscript First-Strand Synthesis System for RT-PCR Kit and oligo(dt) 12–18 (0.5 µg) and random hexamers primers (50 ng). The resulted cDNA was amplified by PCR (35 cycles) using 10 pmoles of each respective primer. PCR amplification of endogenous *Ugcg* mRNA was used as the internal control for RT-PCR reactions. The PCR reactions of each RNA sample without reverse transcriptase were used as negative controls. In the reaction, Primers 1 and 2 were used for GCStg mRNA; Primers 1 and 3 were used for endogenous *Ugcg* mRNA. The primer sequences were as the follows: Primer 1 (EX9fwd): 5-ATTATGCTGTGGCCTGGTTC-3), Primer 2 (EX9UTRrev): 5-TGCTGCAGAGCAGTCCTGTA-3, and Primer 3 (pBroadrev): 5-GACCTCCCACATTCCCTTTT-3. For quantitative analysis, the band intensity of ethidium bromide stained gel was photographed and analyzed using Kodak Digital Science Electrophoresis Documentation and Analysis System (Eastman Kodak Company, Rochester, NY). The values obtained for the transgene band were normalized to the endogenous *Ugcg* bands.

### Fluorescence *in situ* hybridization and Southern blotting

**Figure 1:**
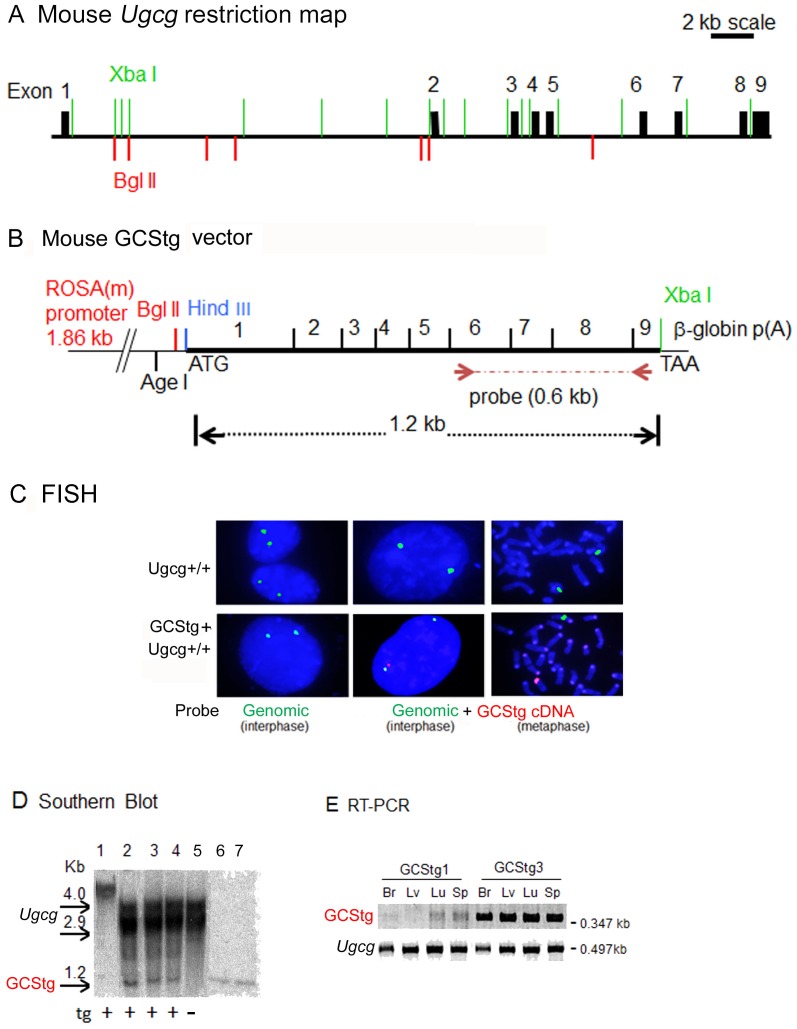
GCS transgene integration and expression. (A) Xba I/Bgl II restriction mapping of the mouse *Ugcg* (GCS) gene. *Ugcg* gene spans 33.5 kb of length and contains 9 exons (solid blocks). The scale bar is 2 kb. (B) The mouse GCS cDNA transgene was cloned into the Hind III/Xba I sites of pBROAD vector containing the ubiquitously expressed ROSA26 promoter and β-globin poly(A) sequence. Arrows show the position of 0.6 kb cDNA probe (exons 6 - 9) for Southern blotting. (C) Fluorescence *in situ* hybridization (FISH) study. Fibroblasts from WT (*Ugcg+/+*) (upper panels) and GCStg mice (lower panels) were processed for FISH analysis using both genomic BAC probe RP23 (green, for endogenous *Ugcg* loci) and GCS cDNA probe (red, for GCStg) as indicated. (D) Southern blot analysis. 20 µg of tail DNA from GCS transgene positive (+) or negative (−) mice were digested by restriction enzymes Xba I and Bgl II and processed for Southern blotting using 0.6 kb [^32^P]-GCS cDNA probe (shown in B). Lanes 1 to 5 from GCStg lines, Lane 1, no Xba I/Bgl II digestion; lane 2 to 5 (GCStg line 1, 3, 9, 10), with Xba I/Bgl II digestion; lane 6 and 7, 1 x or 2 x copy number of 1.2 kb GCS cDNA fragment loaded on the gel. Images of 1.2 kb GCS cDNA bands on Southern blot were quantitated using Image J 1.47V software (NIH, USA). (E) RT-PCR analysis of GCS transgene expression. RNAs from mouse tissues of 2 transgenic mouse lines (GCStg1 and GCStg3) were conducted for RT-PCR using GCStg specific primers and endogenous *Ugcg* primers, respectively. Br, brain; Lv, liver; Lu, lung and Sp, spleen.

Chromosomal fluorescence *in situ* hybridization (FISH) was conducted using fluorescein labeled DNA probes to hybridize metaphase chromosomes and/or interphase nuclei for identifying integration of transgene GCStg in mouse genome. Metaphase chromosome and interphase spread slides were made per standard cytogenetic procedures according to “FISH Procedure 1: For Cell Preparations.” 2010 (Kreatech Diagnostics, Durham, NC). Genomic BAC probe RP23 and GCS cDNA probe (0.6 kb, Fig. 1B) were labeled and *in situ* hybridization was processed according to Nick Translation Kit Labeling Procedure (Abbott Molecular). Southern blotting was conducted as described [43]. Briefly, mouse tail DNA (20 µg) from GCStg PCR-positive lines was digested using Hind III and Xba I, separated on 0.8% agarose gels, and transferred to Hybond N+ nylon membranes. Membranes were hybridized with a [^32^P-dCTP]-labeled 0.6 kb GCS cDNA probe. Hind III and Xba I digested 1.2 kb GCS cDNA fragments were loaded in parallel for quantitation control. The amount of 1.2 kb cDNA fragment which equals to the genome copy number of 20 µg genomic DNA was set as 1 x copies. The developed GCStg Southern bands were quantitated using Image J 1.74V (NIH, USA).

### Preparation of anti-mouse GCS antiserum

A 175 amino acid fragment (aa 66–240) **in the** non-transmembrane coding region of mouse GCS cDNA was cloned into pET21a vector and expressed in BL21 *E.coli* as described [44]. The recombinant GCS peptide (17 kDa) expressed as inclusion body was treated using lysozyme and Triton X-100 and resolved by 10% SDS-PAGE. The signal band (17 kDa) products on polyacrylamide gel were excised to raise rabbit antiserum in Harlan Bioproducts for Science, Inc.

### Western blot

Primary mouse skin fibroblasts were cultured in MDEM-10% BFS with 5% CO_2_ at 37°C [43]. The cell pellets were homogenized in M-PER Mammalian Membrane Protein Extraction Kit and flowed through Detergent Removal Spin Columns to remove detergent in the samples. Protein concentrations were quantified using BCA Protein Assay Reagent and BSA control protein. Tissue extracts were separated on NuPAGE 4–12% Bis-Tris gels and electroblotted on Hybond™-ECL™ PVDF membranes. The membranes were blocked with 5% nonfat dry milk for 1 hr, followed by incubation overnight with rabbit anti-mouse GCS serum (1∶200, Abbiotec) diluted in 1% milk. The signal was developed using ECL detection reagent according to the manufacturer's instructions. The goat anti-rabbit secondary antibody (1∶10,000 in 5% milk) was used to detect mouse GCS and mouse anti-β-actin monoclonal antibody (1∶10,000 in 2% milk) were applied to detect β-actin.

### GCS activity assay

Tissues collected from saline-perfused mice were homogenized in H_2_O and protein concentrations were determined by BCA Protein Assay Reagent. GCS activities were determined fluorometrically with *N*-6-[(7-nitrobenzo-2-oxa-1, 3-diazol-4-yl) amino] hexanoyl-4-d-*erythro*-sphingosine (C6-NBD-ceramide) as described [45]. The incubation system was in a final volume of 125 µL and contained 20 µM C_6_-NBD-substrate complexed with bovine serum albumin (1∶1 molar ratio), 400 µM 1-palmitoyl-2-oleoyl-*sn*-glycero-3-phosphocholine, 400 µM UDP-glucose, 5 mM MgCl_2_, 5 mM MnCl_2_, 1 mM EDTA in 50 mM HEPES (pH 7.2) and tissue homogenate (100–200 µg of protein). After incubation at 37°C for 30 min or overnight, reactions were stopped by the addition of 625 µL (2∶1, v/v) of chloroform-methanol to extract C_6_-NBD lipids in the lower phase. After centrifugation at 1000×*g* for 5 min, the lower phase was evaporated under nitrogen and subjected to HPTLC by using chloroform-methanol-H_2_0 (65∶25∶4, v/v) as solvent. C_6_-NBD lipids present in the chromatograms were visualized using the Storm Imager (Ex 474/Em 530). For quantification, fluorescent spots were then visualized by UV light, spots were scraped, eluted from silica by methanol and then measured using a Shimadzu fluorometer at EX 466 nm/EM 539 nM.

### Tissue lipid analyses

Tissue samples (∼100 mg wet weight) were homogenized in water (0.6 mL) and chloroform:methanol (1∶2, v/v; 3 mL) using a PowerGen 35 (Fisher Scientific). Homogenates were shaken (15 min) and centrifuged (5 min at 1,000×*g*). Pellets were re-extracted with water (0.7 mL) and chloroform-methanol (1∶2, v/v; 3 mL). The combined extracts were centrifuged (10 min at 7,000×g). The supernatants were transferred to fresh tubes and the solvents evaporated under N_2_. Dried extracts were redissolved in chloroform-methanol-water (60∶30∶4.5, v/v/v; 15 mL) and desalted on Sephadex G-25 columns. Samples were then subjected to alkaline methanolysis and desalted. Glycosphingolipids from 4 mg equivalents of tissue samples were quantified by ESI-LC-MS/MS using a Waters Quattro Micro API triple quadrupole mass spectrometer (Milford, MA) interfaced with Acquity UPLC system as described [46], [47].

### Histological Studies

Mice were euthanized in age-matched groups and perfused with 0.9% saline. Brain and visceral tissues (liver, lung, and spleen) were collected, fixed in 10% formalin, embedded in paraffin, sectioned, and stained with hematoxylin and eosin (H&E) then analyzed by light microscopy. For immunohistochemistry, frozen tissue sections fixed with 4% paraformaldehyde were incubated with rat anti-mouse CD68 monoclonal antibody (1∶200 in PBS with 5% BSA). Detection was performed using ABC Vectastain and Alkaline Phosphatase Kit II (black) according to the manufacturer's instruction. The slides were counterstained with methylene green. Images were captured using a Zeiss microscope (Axioskop) equipped with SPOT Advance software (SPOT Diagnostic Instruments, Inc.). CD68-positive macrophages in lung sections were counted manually in randomly selected fields (300 µm×210 µm/field) from non-transgenic and transgenic animals (n = 3 mice/group, 5–15 fields/mouse).

### Immunofluorescence

For cellular localization studies, mouse skin fibroblasts were seeded onto chamber slides one day prior to fixation with 4% paraformaldehyde for 1–2 hrs at room temperature (RT). The fixed cells were washed 3 times with 1X PBS for 10 min each. Then cells were fixed with ice cold methanol (−80°C) for 5 min and washed 3 times with 1X PBS for 10 min each. Cells were then permeabilized with 0.3% Triton X-100/PBS for 1–2 hr RT. Non-specific antibody binding was blocked with 1.5% BSA/1.5% Milk/0.1% Gelatin in 1X PBS 1–2 hrs at RT. The cells were incubated with primary antibodies, rabbit anti-mouse GCS (17 kD) and Alexa Fluor 555 Mouse anti-GM130 (Golgi marker) in blocking solution overnight at 4°C, followed by anti-rabbit conjugated Alexa-Fluor 488 for 4 hrs at RT. The cells were washed with PBS+0.01% Tween 20. Cell nuclei were stained with DAPI. Fluorescence signals were visualized by Zeiss Axiovert 200 M microscopy equipped with an Apotome. Pearson Correlation Coefficients were determined for individual cells using Zeiss Axiovision co-localization software.

### Statistical analysis

The data was analyzed by Student's t-test or one-way ANOVA test.

## Results

### Generation of GCS transgenic (GCStg) mice

The ROSA26 promoter [48] was used to drive ubiquitous GCStg expression (Fig. 1A and 1B). Ten germline-transmitting founder mouse lines were determined by conventional PCR using GCStg specific primers (data not shown). To confirm the chromosomal integration of GCStg, FISH was conducted using interphase and metaphase fibroblasts from GCStg mice. The results showed that endogenous *Ugcg* loci (genomic BAC probe RP23, green) and the GCStg (GCS cDNA probe, red) were at different chromosome locations (Fig. 1C). In comparison, the fibroblasts from WT (*Ugcg*+/+) mice only showed endogenous *Ugcg* loci detected by genomic BAC probe RP23 positive (green). Southern blot analyses showed the presence of a 1.2 kb of Bgl II/Xba I GCS cDNA fragment in 3 GCStg lines (1, 3 and 9) (Fig. 1D, lane 2–4), confirming an intact GCS transgene in the mouse genome. Based upon the density of the 1.2 kb GCS cDNA fragments on the same blot (Fig. 1D, lane 6–7), there were 1–5 copies of GCStg cDNA integrated (Fig. 1D, lane 2–4). Six out of ten germline-transmitting founder mouse lines were analyzed for GCStg expression in different organs. Semi-quantitative RT-PCR was used to estimate the level of GCStg transcripts (Fig. 1E, GCStg) in brain, liver, lung and spleen compared to endogenous GCS mRNA (Fig. 1E, *Ugcg*). GCStg lines showed differential expression levels of transgenic GCS mRNA in brain, liver, lung, and spleen. Among them, the GCStg3 line showed higher levels of GCStg transcripts expression in all tissues tested than the endogenous GCS mRNA (*Ugcg*) (Fig. 1E). In comparison, the GCStg1 line was a low expresser line. The expression of GCStg RNAs was confirmed by Northern blot analysis (data not shown). GCStg3 and GCStg9 lines were backcrossed into *Ugcg+/−,* 9V/9V and 9V/null mice, respectively. Because GCStg3 had high level expression of GCStg, the mice evaluation and analysis were conducted in the *Gba1* mutants, 9V/null and 9V/9V, with GCStg3. The 9V/null/GCStg or 9V/9V/GCStg mice had normal life spans. No abnormal organ sizes and brain pathology were observed. Also, no gross differences were observed in comparison with 9V/null or 9V/9V mice by 1 yr of age.

### GCStg rescues *Ugcg* knockout mice from embryonic lethality

*In vivo* functionality of the GCStg activity was evaluated by first cross breeding transgenic mice with *Ugcg* knockout heterozygotes (*Ugcg*+/−). Subsequent mating provided GCStg/*Ugcg*−/− (transgene/knock-out endogenous GCS) mice that survived embryonic lethality of *Ugcg*−/− [24]. GCStg/*Ugcg*−/− mice were born, thrived, and reproduced with a normal life span, indicating the normal function of transgenic GCS protein *in vivo* and the expression of GCStg at levels that were sufficient to support normal development and function.

### Expression and function of transgenic GCS

**Figure 2:**
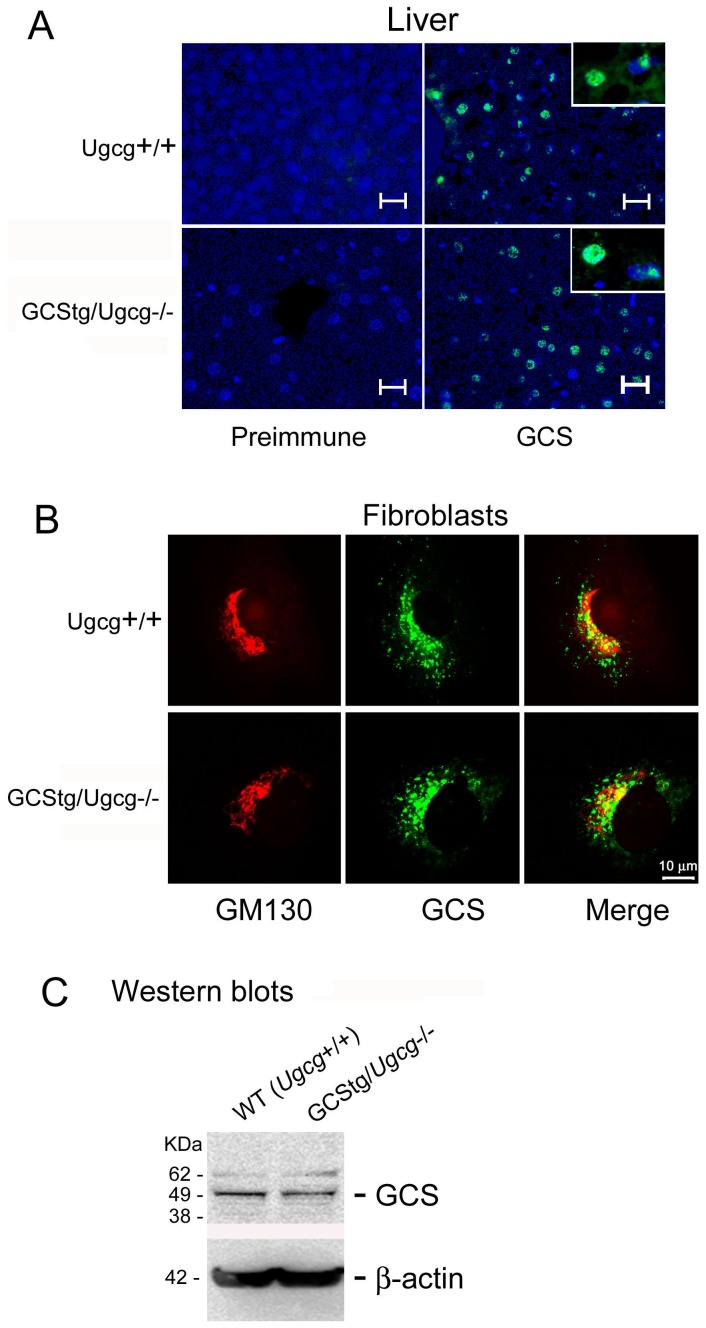
Tissue and cellular distribution of GCStg in mice. (A) Tissue expression of GCStg protein. Frozen liver sections from WT (*Ugcg+/+*) (upper panels) or GCStg rescued *Ugcg*−/− (GCStg/*Ugcg*−/−, lower panels) mice were processed for immunostaining using rabbit anti-mouse GCS antibody (GCS). Inserts show enlarged the cells. Preimmune rabbit serum was used as control. Scale bar  = 10 µm. (B) Cellular localization of GCStg protein. Skin fibroblasts from WT(*Ugcg*+/+) (upper panels) or GCStg (GCStg/*Ugcg*−/−, lower panels) mice in (A) were processed for dual-antibody immunostaining using rabbit anti-mouse GCS (green) and Golgi marker GM130 (red) as indicated. Cell nuclei were stained with DAPI (blue). Pearson Correlation Coefficients (PCC) were determined from individual cells (merged images) using Zeiss Axiovision co-localization software. PCC (*Ugcg*+/+), 43+13% (n = 14 cells); PCC (GCStg/*Ugcg*−/−), 42+12% (n = 19 cells). Scale bar  = 10 µm. Fluorescence signals in (A and B) were visualized by Zeiss Axiovert 200 M microscopy equipped with an Apotome. (C) Western blotting. The fibroblasts in (B) were processed for Western blotting using rabbit anti mouse GCS. Lane 1, *Ugcg*+/+; Lane 2, GCStg/*Ugcg*−/−. β-actin is the loading control.

The protein expression of GCStg was evaluated by immunofluorescence using GCS specific antibody and anti-Golgi antibody GM130 in tissue sections (Fig. 2A) and cultured fibroblasts (Fig. 2B). The results showed transgenic GCS expression in the liver of GCStg/*Ugcg*−/− mice was uniformly distributed around the center sinusoid, which is identical to the WT GCS (*Ugcg*+/+) (Fig. 2A). Similar to WT GCS (*Ugcg+/+*), the transgenic GCS in skin fibroblasts from GCStg/*Ugcg*−/− mice colocalized with GM130 signals showing their Golgi localization. The Pearson Correlation Coefficient was 42+12% in GCStg/*Ugcg*−/− cells, which was not different from 43+13% in WT GCS (*Ugcg*+/+) fibroblasts (Fig. 2B). The expression of GCS in fibroblasts of GCStg/*Ugcg*−/− mice was quantified by Western blotting and the protein level was equivalent to WT GCS in normal mouse fibroblasts (Fig. 2C). These studies showed that the tissue and cellular pattern of GCStg expression was similar to WT GCS.

GCS activity was determined fluorometrically using NBD-ceramide as substrate in GCStg/WT (*Ugcg*+/+) mice tissues. GCS activity was significantly increased in lung and brain, and slightly elevated in liver in GCStg/WT (*Ugcg*+/+) mice compared to WT (*Ugcg*+/+) mice (Table 1). These data indicate that the GCStg has functional GCS activity in tissues.

**Table 1:** GCS activity.

	Lung	Liver	Spleen	Brain
	mean+SEM (n)			
WT	1.90+0.08 (3)	1.35+0.21 (6)	nd	1.00+0.11 (5)
WT+ tgGCS	3.57+0.53 (3)	1.69+0.23 (3)	nd	1.37+0.06 (6)
*p*-value	0.0177	0.181	nd	0.0083

1. GCS activity (nmol NBD-GC/hr/mg) is determined fluorometrically using NBD-ceramide as substrate.

2. *p*-values were from Student's t-test by comparison of WT and WT+tgGCS.

### Transgenic GCS increased substrate concentration in visceral and brain tissues

**Figure 3:**
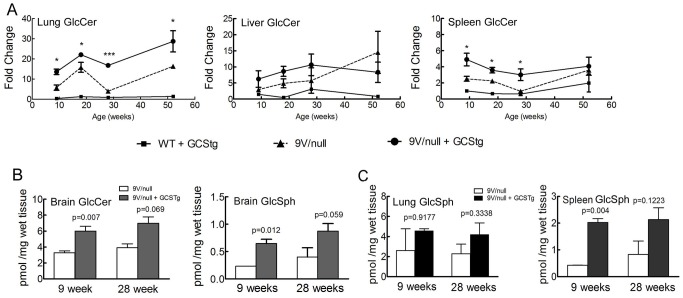
LC/MS analyses of tissue GlcCer and GlcSph concentrations in 9V/null/GCStg mice. (A) GlcCer concentration in visceral tissues. The lungs, livers and spleens from 9-, 18-, 28- and 52-wk of 9V/null (-▴-), 9V/null+GCStg (-•-), WT+GCStg (-▪-) and WT mice were processed for LC/MS analysis. GlcCer concentration in 9V/null, 9V/null+GCStg and WT+GCStg tissues were normalized to GlcCer in WT tissues and present as fold changes (Y-axis) vs. mouse age (weeks) in X-axis. Student's t-test by comparison of 9V/null and 9V/null+GCStg at each age point. *, *p*<0.05; ***, *p*<0.0001. (B) GlcCer and GlcSph concentration in brain. The brains from 9- and 28-wk of 9V/null (open bars) and 9V/null+GCStg (dark bars) mice were processed for LC/MS analysis. GlcCer (left panel) and GlcSph (right panel) concentration present as pmol/mg wet tissue (Y-axis) in bar graph vs. mouse age (weeks) in X-axis. (C) GlcSph concentration in lung and spleen. The lungs and spleens from 9- and 28-wk of 9V/null (open bars) and 9V/null+GCStg (dark bars) mice were processed for LC/MS analysis. GlcSph concentration in lung (left panel) and spleen (right panel) present as pmol/mg wet tissue (Y-axis) in bar graph vs. mouse age (weeks) in X-axis. Experiments were done in triplicate and repeat twice. Results with error bars are mean ±S.E. The *p* values were from Student's t-test by comparison of 9V/null and 9V/null+GCStg.

To increase tissue substrate (GlcCer and GlcSph) load and to accelerate disease phenotypes in the mouse model of Gaucher disease, mice from the GCStg3 founder line were bred into the GCase point mutated 9V/9V and 9V/null mice that had normal WT GCS (*Ugcg*+/+). The resultant mice were termed 9V/9V/GCStg and 9V/null/GCStg. Total GlcCer analyzed by LC/MS showed ∼2-fold increases in concentration in the lungs, livers, and spleens at 9, 18, and 28 wks compared with the respective age matched non-transgenic 9V/null mice (Fig. 3A) and 9V/9V mice (data not shown). By 52 wks, increased GlcCer (2-fold) was present in the lungs, but not in the livers and spleens (Fig. 3A), which suggested a saturation concentration of GlcCer in the livers and spleens with or without the presence of transgenic GCS. GCStg also enhanced GlcCer concentration (2-fold) in the brains of 9V/null mice at 9 wks, which were maintained until at least 28 wks (Fig. 3B). Interestingly, GCStg did increase GlcSph concentration by ∼2 fold in the brains (Fig. 3B), but did not affect GlcSph concentration in the 9V/null liver (data not shown). Increased GlcSph concentrations were detected in the lungs and spleens of 9V/null/GCStg mice, but in the spleen, GlcSph was increased only at 9 wks compared to 9V/null spleen (Fig. 3C). These results indicate that expression of GCStg accelerates GlcCer accumulation at earlier stages in these Gaucher disease mouse models and promotes GlcSph accumulation in the tissues.

**Figure 4:**
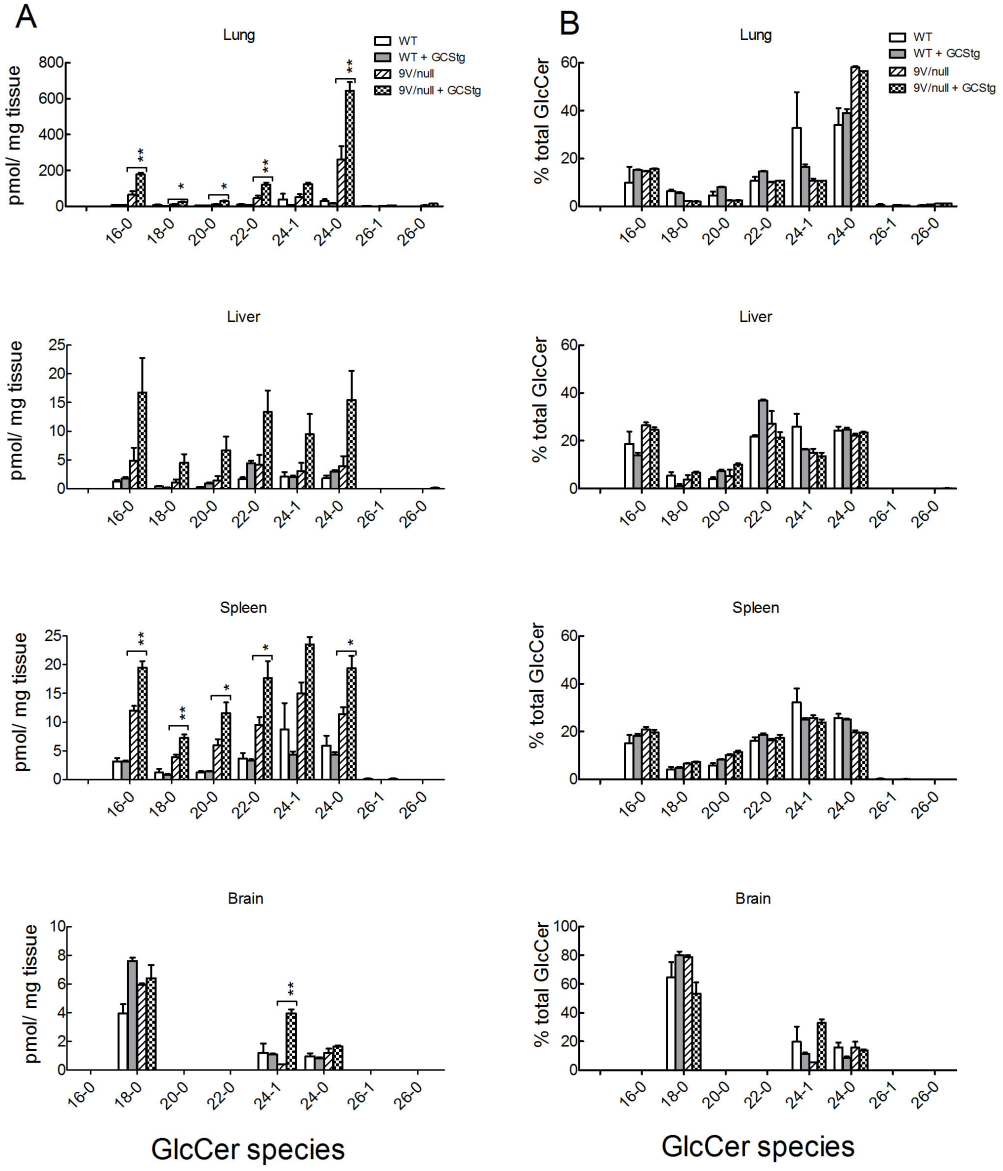
GlcCer species in visceral tissues of 9V/null/GCStg mice. (A) Tissue GlcCer species in the lungs, livers, spleens and brains from 9-wk of WT, WT+GCStg, 9V/null and 9V/null+GCStg mice were quantitated by LC/MS analysis. The concentration of each GlcCer species was plotted as pmol/mg tissue (Y-axis) in bar graph vs. GlcCer species in X-axis. *, *p*<0.05 and **, *p*<0.001 were from one-way ANOVA analysis by comparison of 9V/null and 9V/null+GCStg. (B) Tissue GlcCer species patterns. Each GlcCer species was presented as the percentage of total amount of GlcCer species (Y-axis) in each tissue as indicated. The ratios of each GlcCer species in WT and 9V/null tissues were similar to that in their GCStg counterpart tissues. 9V/null lung had increased GlcCer 24−0 species. Experiments were done in triplicate and repeated at least twice. Results with error bars are mean ±S.E.

In 9-wk 9V/null/GCStg mice, GCStg expression led to increases in all GlcCer species (from 18−0 to 24−0) by 2−3 fold in the lungs, livers and spleens (Fig. 4A). The proportion of 24∶0 was higher in 9V/null and 9V/null/GCStg lungs compared to WT (Fig. 4B) [46]. The relative proportions of GlcCer species were unchanged in 9V/null tissues with GCStg at ages of 18 and 28 wks (data not shown) and 52 wks (Fig. 4B). These data showed the identical function of transgenic and endogenous WT GCSs on synthesis of GlcCer species.

To evaluate the effect of GCS overexpression on the steady state concentration of other sphingolipids, the tissue concentration of ceramide, lactosylceramide (LacCer), and sphingomyelin (SM) were analyzed in WT and 9V/null/GCStg mice. LacCer concentration was slightly increased and SM concentration was slightly decreased in 9V/null/GCStg lungs compared to 9V/null, but the changes were not significant. No major differences in ceramide concentrations were found in 9V/null mice with or without GCStg (data not shown). These results indicate that the GCStg had accelerated effects on GlcCer accumulation in the GCase point mutated 9V/null mice and the effect on other glycosphingolipids is not significant.

### Transgenic GCS increased CD68-positive storage cells

**Figure 5:**
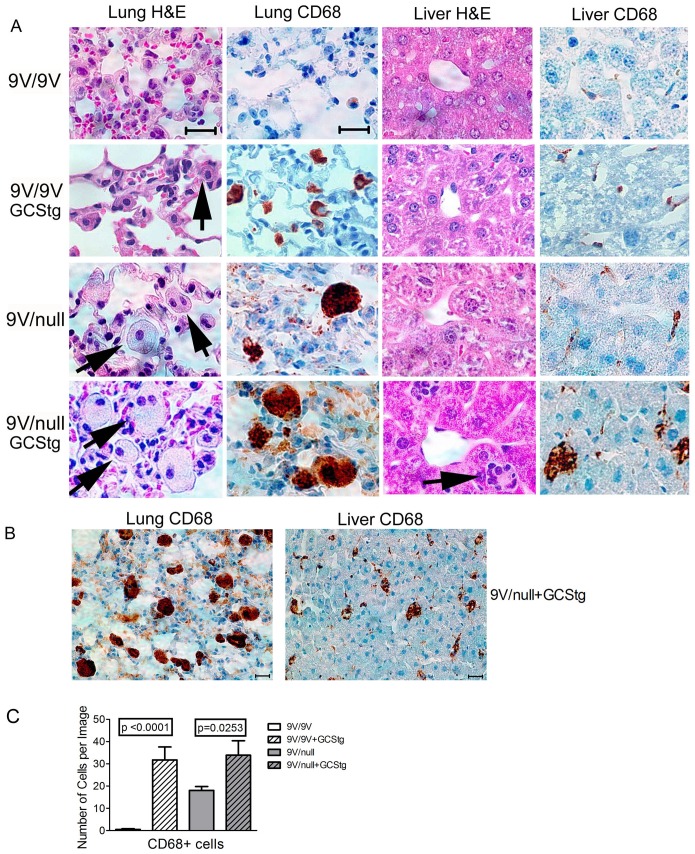
Histology in 9V/9V/GCStg and 9V/null/GCStg mice. (A) The lung and liver sections from 9-wk old 9V/9V (row 1), 9V/9V/GCStg (row 2), 9V/null (row 3) and 9V/null/GCStg (row 4) were processed for H&E and CD68 antibody staining as indicated. Large and pale storage cells were observed in H&E stained lung and liver sections (arrows). The macrophages were indicated by anti-CD68 immunostaining (brown). Images were captured by Zeiss microscope with Spot Advance software. Scale bar was 40 µm for all images. (B) The distribution and density of macrophages in 9V/null/GCStg lung and liver immunostained by anti-CD68 antibody (brown). Scale bar was 40 µm for both images. (C) CD68 positive cells (CD68+) in 9V/9V/GCStg and 9V/null/GCStg lungs had significantly more CD68 stained macrophages than 9V/9V and 9V/null at 9 wks of age, respectively. The data present number of cells per image of total 5−15 images/mouse, 3 mice per genotype. Results with error bars are mean ±S.E. The *p* values were from Student's t-test.

H&E and anti-CD68 stained tissue sections were evaluated for the presence of storage cells, lipid-laden macrophages positive for anti-CD68 antibody, in 9V/null/GCStg mice and 9V/9V/GCStg mice at 9 wks (Fig. 5). In 9V/9V mice, there was neither storage nor CD68-positive macrophages in the lungs and livers (Fig. 5A, row 1). However, in the presence of the GCStg, large numbers of storage cells appeared in the lungs of 9V/9V/GCStg mice (Fig. 5A, row 2). Increased CD68-positive macrophages were observed in the liver of 9V/9V/GCStg compared to that in 9V/9V mice (Fig. 5A, row 2). Similarly, larger numbers and sizes of CD68-positive cells were observed in the lungs and livers from 9-wk 9V/null mice with GCStg (Fig. 5A, row 4) compared with 9-wk 9V/null mice without GCStg (Fig. 5A, row 3). Visual inspection indicated that the size of CD68-positive macrophages is larger in the lung than in the liver suggesting the lung is the most affected organ in the mice (Fig. 5B). Total numbers of CD68 positive storage cells in lung were significantly increased in 9V/9V and 9V/null mice with GCStg at 9 wks of age (Fig. 5C). However, no obvious storage cells were found in the brain of either 9V/9V/GCStg or 9V/null/GCStg mice at these ages. These results demonstrate that increased substrate synthesis by GCStg resulted in GlcCer levels above the threshold which led to rapid phenotype development in visceral tissues of GCase point mutated mice.

## Discussion

In this study, a mouse model carrying a functional GCS transgene was generated to modulate GlcCer concentration *in vivo* and to evaluate tissue substrate threshold in Gaucher disease mouse models, *Gba1* mutant mice. Previously, viable *Gba*1 mutant mice were generated to study the pathophysiologic mechanism in Gaucher disease and therapeutic approaches [14], [33], [49], [50]. However, these mouse models show mild substrate GlcCer accumulation and less severe phenotype compared to their human counterparts [14], [15]. This could be attributed to variations between human and mouse in lipid metabolism. In addition, variations in substrate threshold between human and mouse may account for the difference as well. In Gaucher disease patients, deficient GCase activity leads to substrate accumulation that exceeds the threshold necessary for these patients to develop a phenotype. However, in the mice, except the null models, the substrate threshold in these mutant mice was not reached to develop severe symptoms. Therefore, an integrated GCS transgene could modulate GlcCer production over the threshold for potentiating the phenotypes in *Gba1* point mutant mice. To pursue this hypothesis, a GCS transgene was integrated in *Gba1* mutant mice in this study. The expressed GCStg had the same tissue and cellular distribution as the endogenous GCS protein. Particularly, the GCStg protein was located in the Golgi as the endogenous GCS. Importantly, the integrated GCStg rescued the embryonic lethality of GCS null (*Ugcg−/−*) mice, which demonstrated it has sufficient *in vivo* function. The studies showed that integrated GCStg enhanced GlcCer accumulation and accelerated visceral phenotype development in *Gba1* mutant 9V/9V and 9V/null mice.

Overloading GlcCer could cause accelerated substrate accumulation and appearance of Gaucher-like cells in multiple visceral organs under *GBA*1 normal background. This has been reported in some chronic myelocytic leukemia (CML) patients [51]–[53]. In our unpublished observation, feeding normal macrophages with oxidized red blood cells also led to Gaucher cell-like changes although they were less severe than in 9V/null macrophages. Therefore, the effect of additional or overexpressed GCS on modulation of the GlcCer concentration could promote severe phenotypes in the background of deficient GCase hydrolytic activity. Indeed, the ‘tip of the scale’ by overexpressing GCStg enhanced GlcCer substrate accumulation in 9V/9V and 9V/null mice. The presence of GCStg increased GlcCer concentrations by about 1.5–2 fold in both visceral organs (lung, spleen and liver) and brains compared with their counterpart 9V/9V or 9V/null mice. The excess visceral GlcCer accumulation by GCStg leads to earlier or more severe Gaucher disease histological and biochemical phenotypes. Such tissue GlcCer concentrations could be suggested as the threshold necessary for visceral manifestation in Gaucher disease mice. Within the time frame of this study, the expression of GCStg in brain did not lead to storage cell accumulation and neuronal degeneration in either 9V/9V or 9V/null mice. This may reflect tissue-specific regulation of GlcCer metabolism and GCase activities in the visceral or brain tissues. The underlying mechanism is not clear. Indeed, the residual mutant GCase activities in the brains of 9V/9V or 9V/null mice were more than 5-fold higher than that in the visceral organs [14]. To reach the threshold level in the brain, greatly enhancing GlcCer synthesis or significantly lowering residual GCase activity may be needed. These results suggest that the synthesis and hydrolysis of GlcCer is maintained in lipid metabolism pathway and GlcCer accumulation is mainly controlled by its degradation.

To understand the effect of GCStg expression on other lipid concentrations in the tissues, ceramide, SM, and LacCer were also determined and the results showed no significant changes of ceramide. Cellular ceramide concentration is regulated by enzymatic conversion to other sphingolipids, which provides a tool for intervention [54], [55]. Since the SM was only slightly decreased, the GCStg mainly contribute to the GlcCer production that influences LacCer concentration, but it does not change the cellular ceramide pool. These results indicated that GCS overexpression does not affect cellular ceramide concentration although GCS can convert *de novo* synthesized ceramide to GlcCer [56].

The source of GlcSph in Gaucher disease was verified in this study with Farber patient fibroblasts (data not shown) using the GCS inhibitor C9 and the GCase irreversible inhibitor CBE to confirm GlcSph synthesis occurs via acid ceramidase [42]. Only very little of GlcSph could be detected in acid ceramidase-deficient Farber cells in the presence of CBE, indicating acid ceramidase was the enzyme for GlcSph synthesis by deacylation of GlcCer. These results show that the GCS function mainly contributes to the cellular GlcCer synthesis, and could greatly affect cellular GlcSph concentration by the presence of GCase deficiency. GlcCer can also be made from recycling and salvage of sphingosine to ceramide [57], [58]. The recycling of sphingosine is regulated by phosphorylation and dephosphorylation [58]. This salvage pathway may be predominant in slowly dividing cells [57]. The accumulation of GlcSph and GlcCer in Gaucher diseases could lead to increases of sphingosine that is recycled to more ceramide and complex glycosphinigolipids including GlcCer and GlcSph in specific cell types, e.g. neural cells, which may contribute to tissue/cell type substrates and phenotype variations [59], [60].

Pharmacologically targeting GCS to inhibit GlcCer synthesis and balance the production with impaired degradation is the strategy of substrate reduction therapy (SRT) to treat Gaucher disease [61], [62]. Two SRT drugs have been approved for clinical use, Miglustat (Zavesca, Actelion Pharmaceutical Limited, Switzerland) and Eliglustat tartrate (Genz-112638, Genzyme Corp.) [63], [64]. Miglustat is a synthetic D-glucose analog and Eliglustat is a ceramide analog. They work by inhibiting GCS, thereby reducing endogenous production of GlcCer and complex glycosphingolipids [65], [66]. These SRTs have shown clinical improvement in visceral disease parameters, but have not demonstrated the effects on the neurological deficits [67], [68]. Gastrointestinal disturbances have been reported as the most frequent adverse events associated with Miglustat [69], whereas Eliglustat tartrate was well tolerated [70], [71]. SRT also has implications in other glycosphingolipids storage diseases, e.g Fabry disease and Sandhoff diseases [37], [38], [72]. Our studies of *in vivo* GCS function and tissue specificity in GCase deficient mice demonstrate the cellular GlcCer source is from enhanced GCS activity, thereby supporting clinical application of SRT.

In summary, these studies show that overexpression of GCS leads to enhanced visceral phenotypes in *Gba1* mutant mice. The accelerated storage and lipids accumulation in visceral tissues could provide a shorter testing window for therapeutic evaluations. This study provides an *in vivo* model for threshold substrate flux that modifies phenotypic development in Gaucher disease.
